# Ophthalmic Charts

**Published:** 1892-03

**Authors:** 


*Ophthalmic Charts.* By FRANK HAYDON. London: Down Bros.

These charts will be found of much service in the diagrammatic representation of diseases or abnormalties of the eye. On one set can be drawn the surface appearances of the eyeball; on another what is seen or suspected after a section of the globe. Ophthalmoscopic examination can be represented on a good-sized coloured chart.

